# Measurement of Percentage Depth–Dose Distributions in Clinical Dosimetry: Conventional Techniques and Emerging Sensor Technologies

**DOI:** 10.3390/s26061908

**Published:** 2026-03-18

**Authors:** Giada Petringa, Luigi Raffaele, Giacomo Cuttone, Mariacristina Guarrera, Alma Kurmanova, Roberto Catalano, Giuseppe Antonio Pablo Cirrone

**Affiliations:** Laboratori Nazionali del Sud (LNS), Istituto Nazionale di Fisica Nucleare (INFN), 95123 Catania, Italy; raffaele@lns.infn.it (L.R.); cuttone@lns.infn.it (G.C.); guarrera@lns.infn.it (M.G.); almakurmanova@gmail.com (A.K.); catalano@lns.infn.it (R.C.); cirrone@lns.infn.it (G.A.P.C.)

**Keywords:** relative dosimetry, percentage depth–dose distribution, dosimetry, detectors, radiotherapy

## Abstract

Percentage depth–dose (PDD) distributions are fundamental to characterizing radiation beams in radiotherapy. This review provides an overview of both methods and sensor technologies for measuring PDD in photon, electron, proton, and carbon-ion beams. We summarize conventional dosimetry techniques, including water-phantom scanning with ionization chambers (cylindrical and parallel-plate) and radiochromic film, and discuss their strengths (established accuracy, calibration traceability) and limitations (volume averaging, delayed readout). We then examine emerging sensor technologies designed to improve spatial resolution, speed, and radiation hardness: multi-layer ionization chambers and Faraday cups for one-shot PDD acquisition; scintillator-based detectors (liquid, plastic, and fiber-optic) enabling real-time and high-resolution depth–dose measurements; advanced semiconductor detectors including silicon carbide diodes; as well as novel approaches such as ionoacoustic range sensing for proton beams. For each modality and detector type, we emphasize clinical relevance, measurement accuracy, spatial resolution, radiation durability, and suitability for high dose-per-pulse environments (e.g., FLASH radiotherapy). Current challenges, such as detector response in regions of steep dose gradient, saturation or recombination at ultra-high dose rates, and energy-dependent sensitivity in mixed radiation fields, are analyzed in detail. We also highlight the limitations of each technique and discuss ongoing improvements and prospects for clinical implementation. In summary, no single detector technology fully satisfies all requirements for fast, high-accuracy, high-resolution, radiation-hard PDD measurement, but the integration of emerging sensor innovations into clinical dosimetry promises to enhance the precision and efficiency of radiotherapy quality assurance.

## 1. Introduction

Accurate measurement of percentage depth–dose (PDD) distributions in water is a pillar of radiotherapy beam commissioning and quality assurance. The PDD curve describes the relative dose delivered along the beam central axis as a function of depth in a water phantom, typically normalized to a reference level (most often the dose maximum), and provides a compact representation of how energy is deposited in a tissue-equivalent medium. In clinical practice, PDD datasets are routinely used for beam calibration, for the configuration and validation of treatment planning system (TPS) beam models, and for periodic quality assurance (QA) procedures, ensuring that the delivered dose matches the prescription and that the dose gradients required to spare surrounding healthy tissues are maintained. Consistently, international reference dosimetry protocols incorporate PDD-derived beam-quality indices as key parameters linking the measured detector signal to absorbed dose to water. Two of the most widely adopted protocols are the AAPM TG-51 protocol and the IAEA TRS-398 Code of Practice, which define standardized procedures for reference dosimetry in external-beam radiotherapy using ionization chambers under well-defined reference conditions [1,2]. Because these indices and depth–dose descriptors propagate through both absolute and relative dosimetry, PDD measurements must achieve high precision across modalities, from photons and electrons to protons and heavier ions, to ensure robust commissioning decisions and reproducible long-term beam performance [1]. In clinical photon and electron beams, uncertainties in the build-up region and in the distal fall-off affect reference-depth definitions and the validation of treatment planning system depth scaling, while in charged-particle beams the clinically relevant information is tightly linked to the depth of the Bragg peak/distal edge, so that millimeter-level errors in depth assignment or range determination can translate into clinically relevant shifts of the high-gradient region [3]. Given that overall delivery tolerances are typically on the order of a few percent, reducing PDD-related uncertainties remains a practical driver for both detector development and measurement protocols [1]. Water-phantom scanning with ionization chambers remains the benchmark approach because it provides a robust and traceable route to dose-to-water measurements; however, it is intrinsically time-consuming and can be limited by volume averaging in steep gradients, as well as by collection-efficiency issues when dose-per-pulse increases, which may bias readings particularly where gradients are most pronounced (e.g., photon build-up, electron fall-off, and particle-beam distal edges) [4]. High-resolution passive techniques such as radiochromic films mitigate spatial averaging and can resolve fine PDD features, but they typically require careful handling and post-processing (scanning, calibration, and stabilization time), and they are therefore less suited to fast iterative workflows or true real-time use [5]. These practical limitations become more stringent as modern delivery techniques push toward smaller fields, tighter margins, and higher instantaneous dose rates, including ultra-high dose-rate (UHDR) scenarios where conventional dosimeters may deviate from ideal linearity or require non-trivial corrections [6]. Consequently, recent research has moved toward complementary strategies that trade off absolute traceability, spatial resolution, and speed in a controlled way: multi-sensor and multi-layer concepts that acquire the depth–dose curve (or a clinically relevant surrogate) in a single irradiation; compact solid-state detectors with small sensitive volumes and radiation-hard materials; scintillator-based systems that enable rapid readout; and non-invasive range-verification approaches that aim to provide online, measurement-driven constraints on depth–dose and range during delivery [7,8]. In this work, we first outline the key features of PDD curves in photon, electron, proton, and carbon-ion beams, emphasizing how the underlying interaction physics and clinical delivery conditions translate into modality-specific dosimetric challenges. We then review, in a structured manner, both established dosimeters used in routine practice (e.g., cylindrical and parallel-plate ionization chambers, films) and emerging detector concepts developed to address current limitations (e.g., multi-layer devices, scintillator-based systems, semiconductor diodes, fiber-optic sensors, and acoustic approaches). For each detector family, we summarize the operating principle and practical implementation for depth–dose acquisition, and we critically discuss the achievable accuracy and longitudinal resolution as well as robustness under clinically relevant conditions, including the adaptations and corrections typically required in demanding scenarios such as small fields or UHDR delivery. A dedicated comparative section then benchmarks the different technologies against a common set of performance metrics, including spatial resolution, capability for real-time or near-real-time readout, dynamic range, and radiation hardness, with the aim of supporting an informed selection of measurement tools for specific applications. Finally, we discuss open challenges and future directions in PDD dosimetry, highlighting how advances in measurement speed, precision, and robustness can reduce range- and model-related uncertainties, enable tighter treatment margins, and ultimately improve the therapeutic ratio by better sparing normal tissue while maintaining target coverage [2,9].

## 2. PDD Characteristics in Different Radiotherapy Modalities

### 2.1. Photon Beams

**Figure 1:**
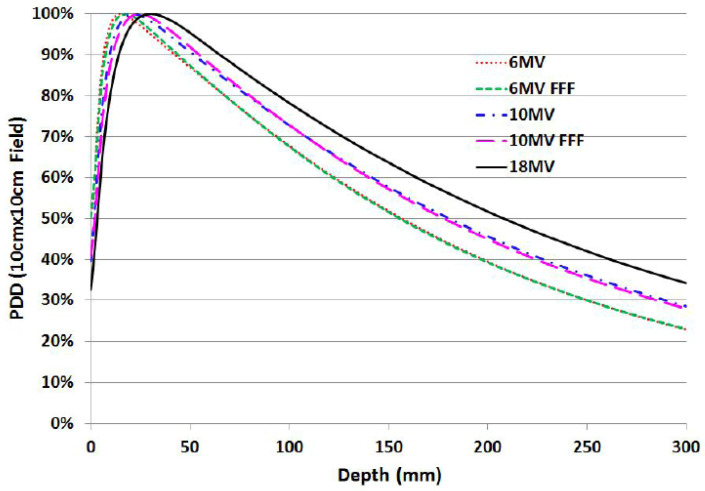
PDD curves of 6 MV, 6 MV FFF, 10 MV, 10 MV FFF, and 18 MV photon beams for a 10×10 cm^2^ field at 100 cm SSD [11].

Megavoltage photon beams (generated by medical linear accelerators) exhibit PDD curves with a characteristic surface-sparing build-up region followed by a quasi-exponential attenuation with depth. The PDD curve, defined as the relative dose versus depth along the beam central axis in water (or tissue-equivalent media) and normalized to a reference value (often the dose maximum), provides a compact descriptor of beam penetration and of the conditions for (transient) charged-particle equilibrium in the first centimeters of depth [10]. Representative PDDs for 6 MV, 10 MV and 18 MV beams, comparing flattened and flattening-filter-free (FFF) modalities for a 10×10 cm^2^ field at 100 cm source-to-surface distance (SSD), are shown in Figure 1.

In Figure 1, the progressive shift of the depth of maximum dose $d_{\max}$ with increasing nominal energy (e.g., ∼1.5 cm for 6 MV under reference conditions) and the increased penetration at larger depths are clearly observed [10]. Modest differences between flattened and FFF beams can arise from changes in head scatter, spectrum and dose-per-pulse characteristics, and must be considered when selecting detectors and applying corrections under clinical commissioning conditions [12,13].

Key features of photon PDDs include:

- **Surface dose and build-up:** the dose rises steeply from the surface to $d_{\max}$ as secondary electrons build up toward (transient) charged particle equilibrium; beyond $d_{\max}$ the PDD decreases approximately exponentially with depth due to attenuation and scatter [10].
- **Electron contamination:** contaminant electrons generated in the linac head (e.g., flattening filter/collimators) and in air increase the surface dose and can reduce the apparent steepness of the build-up. This contribution generally increases with field size and decreases with SSD, and is energy- and geometry-dependent [14].
- **Dosimetric indices/beam quality:** clinical beam quality is commonly specified through depth–dose metrics such as $\%dd{\left(10\right)}_{x}$ (often denoted $PDD{\left(10\right)}_{x}$) in TG-51, or via $TPR_{20/10}$ in TRS-398. These indices enter reference dosimetry through quality-conversion factors (e.g., $k_{Q}$) and require consistent reference conditions and detector corrections [1,15,16].

Accurate measurements in the build-up region remain challenging because steep gradients and a lack of full equilibrium amplify detector perturbations and effective point-of-measurement effects. Plane-parallel chambers are often preferred at shallow depths, but fixed-separation plane-parallel chambers may exhibit over-response in the build-up region; therefore, chamber-specific correction strategies and careful experimental methodology are required [17,18]. For small or highly modulated fields (where volume averaging and perturbation effects become critical), additional guidance on detector choice and correction factors is provided by TRS-483 [19].

In practice, photon PDDs are typically acquired in scanning water phantoms using cylindrical ionization chambers or diodes for the descending part of the curve, with dedicated approaches (small-volume detectors, plane-parallel chambers, or alternative dosimeters) for the build-up. High dose-per-pulse conditions—particularly relevant for FFF beams—require appropriate ion recombination corrections and attention to potential dose-rate dependencies [1,12]. Advanced dosimeters such as plastic scintillation detectors can provide near water-equivalent, high-spatial-resolution measurements for surface and build-up dosimetry, reducing volume averaging in steep gradients [20].

Finally, unlike charged-particle beams, photon PDDs exhibit a long attenuation tail, implying non-zero dose at large depths and contributing to integral dose outside the target; this is one of the physical motivations behind the use of proton and heavier-ion beams to reduce distal dose deposition [2].

### 2.2. Electron Beams

**Figure 2:**
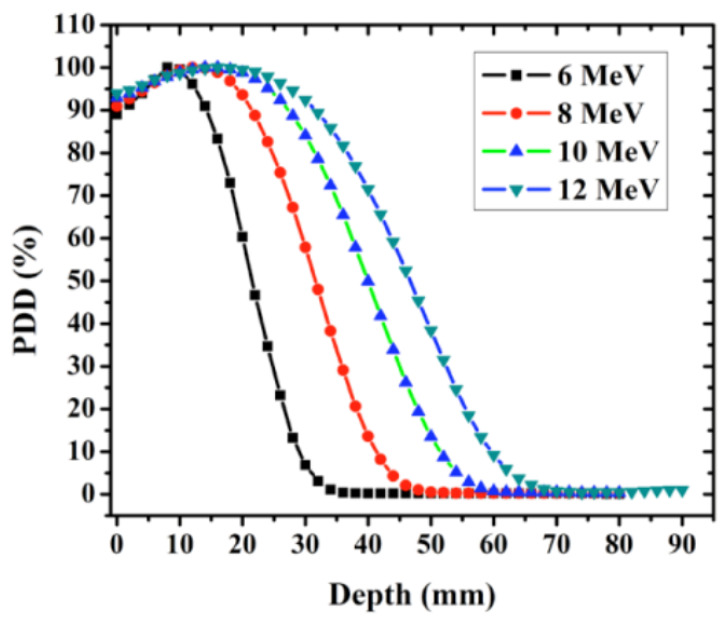
Central-axis PDD curves in water for 6, 8, 10 and 12 MeV clinical electron beams; curves are normalized to the dose maximum [22].

Clinical megavoltage electron beams exhibit PDD curves that markedly differ from photon beams, reflecting the finite range of charged particles in matter. Electron PDDs are characterized by a relatively high surface dose, a shallow depth of maximum dose $d_{\max}$, and a broad high-dose region followed by a rapid distal fall-off approaching the practical range. As illustrated in Figure 2, the penetration depth increases with nominal energy (here 6, 8, 10 and 12 MeV), while the dose beyond the fall-off is dominated by a low-amplitude bremsstrahlung tail (photon contamination) originating from the linac head and from interactions in the phantom [21].

Key features of electron PDDs include:

- **Surface dose and shallow build-up:** because the primary radiation is charged, electron beams deliver a comparatively high entrance dose and reach $d_{\max}$ within the first millimeters to centimeters, depending on energy and applicator/cutout conditions.
- **Finite range and steep distal gradient:** the sharp dose drop near the end of range underpins the clinical suitability of electrons for superficial targets, but makes the distal region sensitive to energy, SSD, field size, and lateral scatter equilibrium.
- **Dosimetric indices/beam quality:** electron beam quality is specified using $R_{50}$ (depth in water where absorbed dose falls to 50% of its maximum under reference conditions). In TRS-398, the mean energy at the phantom surface is often approximated as
  (1) $$E_{0}≃2.33\;R_{50},$$
  with $E_{0}$ in MeV and $R_{50}$ in cm. In TG-51 reference dosimetry, the reference depth is defined as
  (2) $$d_{\mathrm{ref}}=0.6\;R_{50}-0.1\;\mathrm{cm},$$
  linking beam quality to calibration conditions and quality-conversion factors.

In clinical practice, electron PDDs are measured in scanning water phantoms using ionization chambers and/or solid-state detectors. For accurate characterization near the surface and in the high-gradient fall-off region, detector choice is critical: plane-parallel chambers are commonly preferred for shallow depths, while small-volume cylindrical chambers, diodes, films, or plastic scintillation detectors can be advantageous when high spatial resolution is required (e.g., small fields, steep gradients) [1,15,20]. As in the case for photon beams, consistent commissioning requires attention to gradient/volume-averaging effects, effective point-of-measurement conventions, and—when operating at high dose-per-pulse—appropriate ion recombination corrections [1,16].

### 2.3. Proton Beams

**Figure 3:**
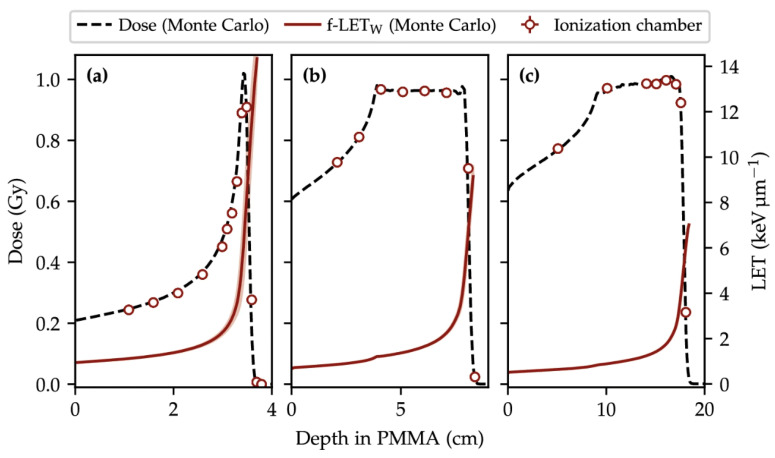
Examples of proton depth–dose (dashed black, Monte Carlo) and fluence-averaged LET in water *f*-LET_*w*_ (solid red, Monte Carlo) as a function of depth in PMMA; red markers represent ionization-chamber measurements. Panel (**a**) shows a monoenergetic 70 MeV proton beam (single energy layer), while panels (**b**,**c**) correspond to SOBP configurations used to produce a uniform dose over a finite depth range. The curves highlight the steep distal fall-off in dose and the marked increase of LET toward the end of range [23].

Proton therapy beams produce a Bragg curve (depth–dose distribution) that is fundamentally different from photon PDDs: the dose is relatively low in the entrance region, increases with depth, and culminates in a sharp Bragg peak close to the end of the particle path, followed by a rapid drop-off to (nearly) zero dose beyond the range. This characteristic behavior is illustrated in Figure 3, which also highlights the concomitant increase of Linear Energy Transfer (LET) with depth, with the highest LET values occurring near the distal edge/Bragg peak region.

Key aspects of proton PDD measurements include:

- **Stopping power and water-equivalent thickness (WET):** accurate depth assignment requires mapping the measurement depth to water-equivalent thickness, especially when range shifters, slabs (e.g., PMMA), or heterogeneous materials are present.
- **Range and output QA:** range metrics derived from the distal edge (e.g., $d_{90}$, $R_{80}$, distal fall-off width) are core quantities for commissioning and periodic quality assurance.

For a quasi-monoenergetic proton beam, the entrance dose slowly increases and reaches its maximum at the Bragg peak (Figure 3a). In parallel, LET rises modestly in the entrance/plateau and then increases steeply toward the range (red curve in Figure 3), reflecting the progressive energy loss and the higher stopping power at low proton energies. Immediately beyond the peak, the dose falls to nearly zero within a few millimeters (distal fall-off). Because clinical treatments exploit this finite range to spare tissues beyond the target, sub-millimeter range reproducibility is critical; improved range accuracy directly reduces the safety margins needed around the tumour [3]. In clinical practice, a Spread-Out Bragg Peak (SOBP) is created by superimposing multiple Bragg peaks of different energies to deliver an approximately uniform physical dose over the tumor volume. The SOBP therefore exhibits a high-dose plateau and a distal fall-off at the end of the modulation (Figure 3b,c). In routine QA (e.g., AAPM TG-224 [3]), depth–dose/range constancy checks are commonly performed using ionization chambers in water as reference instruments, often complemented by higher-resolution detectors (diodes, scintillators) for accurate distal-edge localization. A persistent experimental challenge is reconciling detector readings in high-gradient/high-LET regions: finite sensitive volumes can smear the Bragg peak, and ion recombination and LET-dependent response can bias dose measurements near the peak, requiring careful detector selection and corrections [4,24]. Multi-layer ionization chambers (MLICs) and scintillator arrays can capture the entire Bragg curve in a single irradiation, enabling fast verification workflows in modern pencil-beam scanning systems [25,26]. In parallel, solid-state detectors (e.g., silicon, diamond, SiC) with sub-millimeter sensitive volumes are increasingly investigated to improve spatial resolution and robustness, particularly for advanced delivery modes and ultra-high dose-rate conditions [27,28,29,30,31,32].

### 2.4. Carbon-Ion Beams

Carbon-ion radiotherapy is characterized by even higher-LET particles than protons, resulting in a Bragg peak with a pronounced fragmentation tail beyond the peak [2]. When carbon ions penetrate tissue, nuclear interactions produce lighter fragments (e.g., helium, lithium ions) that continue past the primary range and deposit residual dose after the primary Bragg peak [33,34]. Accurately measuring the Bragg peak height and the tail region is important for treatment planning, as the tail contributes to out-of-target dose and can influence normal tissue exposure distal to the tumour. Standard detectors (ion chambers, diodes) can generally capture the broad features, but capturing fine structure may require high-resolution detectors or chemical dosimeters (e.g., gels). A key consideration for carbon-ion PDD measurements include the detector quenching at high LET [35,36]. Many detectors exhibit LET-dependent response. For example, plastic scintillators and organic scintillating screens suffer quenching, a reduced light yield per unit dose, in the high-LET regions near and beyond the carbon Bragg peak. This means that a plastic scintillator might underestimate the dose in the peak and tail region of carbon beams [35,36]. Similarly, radiochromic film can saturate or under-respond in high-LET regions [37,38,39]. To address this, detectors must be calibrated in ion beams or corrected empirically (e.g., using Birks-like quenching curves) if used for carbon PDD.

Parallel-plate ion chambers (e.g., large-area Bragg peak chambers) are often employed for carbon depth–dose measurements because they provide a stable integrated reading of Bragg peak depth. However, as with protons, recombination and volume averaging can be significant [40,41,42]. Multi-layer ionization chambers, such as those used in proton QA, have also been tested with carbon beams, but high LET introduces additional complications (increased recombination, gas-ionization cluster effects) [26]. Diamond and silicon carbide detectors have been investigated for carbon-beam dosimetry due to their radiation hardness and LET resilience [28,43,44]. Matsumoto et al. (2023) developed a SiC-based dosimeter to evaluate dose distributions in a clinical carbon beam, finding stable performance and the ability to infer LET spectra [44]. These studies indicate that wide-bandgap semiconductors can operate in the intense radiation fields of carbon therapy without significant damage or signal drift, in contrast to conventional silicon diodes which might suffer damage from the high neutron and secondary-particle background. Overall, carbon-ion PDD measurement leverages many of the same tools as proton dosimetry, but the higher LET and fragmentation dose require careful detector choice and calibration. Diamond detectors have been used in carbon beams due to their near-tissue equivalence and relatively low LET dependence [45,46,47]; synthetic diamond probes can measure depth–dose with fine resolution and minimal quenching [38,48]. Multi-layer Faraday cups and ionization chambers have also been used in research to measure the energy spectrum of carbon beams, which indirectly provides depth–dose information via range-energy relationships [49,50].

## 3. Conventional PDD Measurement Techniques and Detectors

### 3.1. Water Phantoms and Ionization Chambers

Scanning ionization chambers in a water phantom have long been the reference method for acquiring PDD curves for external clinical beams. The water phantom provides a tissue-equivalent medium, and a detector is translated to various depths to record dose [51,52]. A depth–dose scan is typically performed by positioning the chamber at incremental depths (using an automated motor or manual adjustment) and measuring the collected charge at each point, normalized either to the maximum value or to a reference depth. Ionization chambers do not measure absorbed dose directly: the primary quantity acquired in a water-tank scan is a *depth–ionization distribution* (PDI), i.e., the chamber reading M(z) (charge/current) as a function of depth *z*. Converting PDI to a *depth–dose distribution* (PDD) requires accounting for the water-to-air stopping-power ratio, which may vary with depth depending on the modality. In relative form, the conversion can be written as [1]:

(3) $$\frac{D(z)}{D(z_{\mathrm{ref}})}\approx \frac{M\left(z\right)\;s_{w,\mathrm{air}}\left(z\right)}{M\left(z_{\mathrm{ref}}\right)\;s_{w,\mathrm{air}}\left(z_{\mathrm{ref}}\right)}\;,$$

where $z_{\mathrm{ref}}$ is a chosen normalization depth. For megavoltage photon beams, $s_{w,\mathrm{air}}$ is often approximately constant (to good accuracy) at and beyond $d_{\max}$, so relative ionization can be used as a proxy for relative dose in that region. For electron, proton and ion beams, instead, $s_{w,\mathrm{air}}\left(z\right)$ varies with depth and the PDI→PDD conversion should be applied explicitly. Unless otherwise stated, in the following we use the term PDD to indicate the absorbed-dose curve in water; when ionization chambers are used, PDD curves are obtained from the measured PDI according to Equation (3). Modern scanning systems allow depth steps as small as 0.1 mm when required [53].

**Figure 4:**
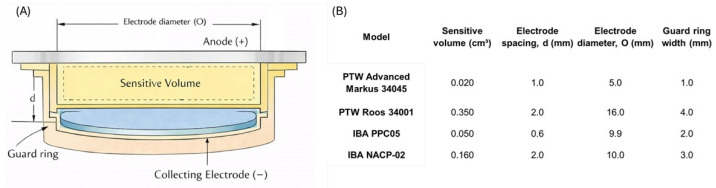
(**A**) Schematic cross-sectional view of a parallel-plate ionization chamber. The sensitive volume is defined by the gas region between the high-voltage anode and the collecting electrode (cathode), while a guard ring, held at the same potential as the collecting electrode, is used to minimize edge effects and leakage currents. The electrode diameter, O, and the electrode spacing, d, are indicated. (**B**) Geometrical characteristics of selected commercial parallel-plate ionization chambers, including sensitive volume, electrode spacing, electrode diameter, and guard ring width [1,58,59,60,61].

During scanning, a reference detector placed at a fixed point in the field is often used to correct for beam output fluctuations, especially for photon beams. Different ionization-chamber geometries are employed depending on beam type and measurement region. Cylindrical (thimble-type) chambers, such as Farmer-type chambers (sensitive volume ≈0.6 cm^3^), are widely used for PDD measurements of megavoltage photon beams [1,15,54]. Smaller-volume cylindrical chambers (e.g., pinpoint chambers of 0.01–0.1 cm^3^) are preferred when finer spatial resolution is required or when characterizing smaller treatment fields [19,55,56,57]. Parallel-plate ionization chambers constitute a specialized subclass, featuring a thin, wide geometry with two flat electrodes separated by a small gap (typically 1–2 mm). Examples include the PTW Markus, NACP-02, IBA PPC-05, and Roos chambers (see Figure 4) [1,58,59,60,61].

For central-axis depth–dose work, IAEA TRS-398 (Rev. 1, 2024) [1] specifies modality- and region-dependent conventions that should be made explicit when reporting PDD curves:

- **High-energy photons:** plane-parallel ionization chambers are recommended for acquiring depth–dose curves. If a cylindrical chamber is used (Farmer/pinpoint), the effective point of measurement must be accounted for by shifting the full depth–ionization curve toward the surface by $0.6\;r_{\mathrm{cyl}}$, where $r_{\mathrm{cyl}}$ is the cavity radius.
- **High-energy electrons:** for all beam qualities, the preferred detector for depth–dose distributions is a plane-parallel chamber. A cylindrical chamber may be used for $R_{50}>3\;g/{\mathrm{cm}}^{2}$ (approximately $E_{0}>8$ MeV), with the reference point positioned at the appropriate depth (deeper than the point of interest) following protocol conventions.
- **Protons and ions:** plane-parallel chambers are recommended for depth–dose measurements. For pencil-beam scanning (PBS) delivery systems, pencil-beam depth–dose measurements should be carried out with large-area plane-parallel chambers (up to ∼150 mm cavity diameter) so that the full non-scanned beam diameter, including scattered protons/ions, is integrated; the resulting curve represents an integrated radial profile as a function of depth.

These protocol choices and positioning conventions are tightly connected to reference dosimetry: ion-chamber measurements underpin beam-quality indices such as PDD(10) or TPR_20/10_ in photon beams and the reference conditions used for electron and ion beams [1,15,16,52,62]. Therefore, even as new detector technologies emerge, careful adherence to ion-chamber handling and correction procedures in water phantoms remains central to clinical dosimetry.

#### 3.1.1. Advantages

Ionization chambers provide a calibrated, stable response that, after applying the appropriate calibration and correction factors, is proportional to the absorbed dose to water. When used with appropriate chamber types and beam-quality dependent calibration factors, their response is nearly energy-independent for megavoltage photon beams and high-energy proton beams, making them suitable as primary reference instruments across a wide range of clinical conditions [1,52,63]. Their signal exhibits minimal depletion, so chambers can be reused for long periods and still maintain reliable performance, with immediate readout via standard electrometers. The finite sensitive volume, which is typically 3–7 mm long in Farmer-type chambers, can sometimes be beneficial, as the associated volume-averaging smooths high-frequency noise or small-scale fluctuations in beam microstructure, improving signal stability in routine QA measurements [55,64,65]. The combination of a water phantom and a scanning ionization chamber enables direct measurement of depth–dose distributions in a medium that closely approximates soft tissue. In photon-beam dosimetry, this approach allows accurate determination of key beam-quality indices such as PDD(10) or TPR_20/10_, which are essential for absolute calibration according to protocols like AAPM TG-51 and IAEA TRS-398. Scanning systems can be configured with very fine step sizes (down to 0.1 mm), enabling detailed reconstruction of PDD curves where gradients are moderate and chamber size is compatible with the desired resolution. Parallel-plate ionization chambers provide additional advantages in situations where the dose is highest near the surface or where steep gradients occur at shallow depths. In conventional electron therapy (6–20 MeV), they are considered the detectors of choice for PDD measurements: the thin entrance window can be placed flush with the phantom surface, minimizing disturbance in the first millimeters and enabling accurate measurements of surface dose and build-up [1,62]. This is why IAEA and AAPM protocols recommend plane-parallel chambers for low-energy electrons, where cylindrical chambers become less reliable [24]. Similar considerations apply to the build-up region of high-energy photon beams, where the large cross-sectional area of parallel-plate chambers ensures uniform sampling of the beam profile, and the minimal perturbation thickness provides more reliable surface-dose readings than Farmer-type chambers. In proton dosimetry, parallel-plate chambers such as the Markus have been widely used as reference detectors for depth–dose measurements due to their well-defined geometry, small effective cavity thickness, and known perturbation factors [24,66]. When placed at successive depths in a water phantom, they can map the full Bragg peak with good reproducibility and are often used in cross-comparisons with other detector systems (e.g., silicon diodes, multi-layer devices). Studies by Medin et al. and Palmans et al. have shown that, when appropriate calibration and correction factors are applied, plane-parallel and cylindrical chambers yield consistent proton ranges and depth–dose characteristics [4,24,66]. Over the past decades, ionization chambers in general have proven robust and reliable in both proton and carbon-ion beams, consolidating their role as benchmark detectors for clinical reference dosimetry and QA [1,2,63]. Ionization chambers are also indispensable for cross-calibrating and validating alternative detectors with higher intrinsic spatial resolution. A common strategy in both photon and proton dosimetry is to use an ion chamber (e.g., a Farmer or Markus device) to establish the absolute dose at a reference point on the PDD curve, and then to employ detectors such as solid-state diodes, scintillators, or radiochromic films to map the fine details of the curve shape. In this way, the strengths of ion chambers (absolute accuracy and traceable calibration) are combined with the spatial resolution and real-time capabilities of newer technologies [5,55,67].

#### 3.1.2. Limitations

Despite their widespread use, ionization chambers present intrinsic limitations that can impact the accuracy of PDD measurements, particularly in regions of steep dose gradient. The finite size of the sensitive volume leads to spatial averaging: in the photon build-up region or in the Bragg peak of proton beams, a Farmer-type chamber can underestimate the true peak dose and overestimate the width of sharp features, effectively smoothing the distal fall-off and plateau–peak transitions [4]. This spatial blurring becomes more pronounced as the gradient increases relative to the chamber’s cavity length. While smaller-volume cylinders and thin plane-parallel chambers mitigate this effect, they do not entirely remove it, especially when the gradients occur over sub-millimetric distances. Ion recombination within the chamber cavity is another important source of limitation. At conventional dose rates, well-designed chambers typically exhibit recombination losses of only a few percent or less, which can be corrected using established two-voltage methods or Boag-type recombination formulas [1,16,68,69]. However, in beams with high dose-per-pulse, such as pulsed proton beams or ultra-high dose rate (FLASH) conditions, ion recombination can become substantial and lead to a marked under-response if not carefully corrected [4,6]. The water-phantom scanning technique itself is relatively time-consuming and operationally demanding. A full set of PDD curves for different field sizes and energies may require multiple scans, each taking several to tens of minutes, and the setup involves precise alignment, periodic checks of water level and temperature, and careful handling of cables and positioning systems. For proton-beam commissioning, acquiring multiple Bragg curves with a single scanning chamber can therefore be laborious, which has motivated the development of faster multi-layer devices. Additionally, although modern scanning systems allow step sizes as small as 0.1 mm, the combination of chamber-size averaging and mechanical step resolution still imposes a practical limit on the longitudinal resolution achievable with ionometric techniques. Another practical factor affecting the accuracy of PDD measurements is the precision of detector positioning within the water phantom. In regions characterized by steep dose gradients, such as the photon build-up region, the distal fall-off of electron beams, or the Bragg peak of particle beams, even sub-millimetric positioning uncertainties can translate into measurable deviations in the reconstructed depth–dose curve. Although modern scanning systems provide high mechanical precision, the effective measurement point of the detector, alignment procedures, and operator handling may introduce additional uncertainties. For this reason, careful detector positioning and consistent measurement protocols are essential to ensure reproducible PDD measurements in routine clinical practice. Ionization chambers also perturb the radiation field to some extent. Their air cavity, electrode materials, and housing introduce a departure from charged-particle equilibrium and modify the local fluence. For megavoltage photons and high-energy protons, these perturbations are relatively small and have been extensively quantified, but at lower energies and near the surface more substantial corrections are required. Reference dosimetry protocols such as IAEA TRS-398 and AAPM TG-51 provide beam-quality-conversion factors that relate the chamber reading in a beam of interest to the calibration in a ^60^Co or reference beam, thereby accounting for differences in energy spectrum, geometry, and perturbation effects [2]. For relative PDD measurements, absolute calibration is less critical, but it remains essential to verify that the chamber operates in its linear regime (without significant polarization or recombination artifacts) and that any residual energy dependence is consistent along the depth scan. Parallel-plate chambers, while indispensable in high-gradient regions, introduce their own specific limitations. Their larger electrode surfaces and small gap make them more sensitive to mechanical tolerances and handling; they can be somewhat more delicate than standard cylindrical chambers and may exhibit pronounced polarity effects or increased leakage currents if not properly maintained. They must be carefully calibrated, often in a ^60^Co or high-energy electron beam, to ensure accurate use in electron and proton fields [24]. The effective point of measurement is located at the inner surface of the entrance window and must be explicitly accounted for when positioning the chamber in the water phantom. Failure to apply the correct effective-depth shift can introduce systematic errors in reconstructed PDD curves. Altogether, these factors mean that while ionization chambers in water phantoms remain the benchmark for PDD measurements and reference dosimetry, they are not free from systematic and practical limitations. Their finite sensitive volume, recombination effects, beam perturbation, and the time-consuming nature of water-phantom scanning motivate the exploration of complementary or alternative detector systems.

### 3.2. Radiographic and Radiochromic Film Dosimetry

Film dosimetry has long been a pillar for measuring two-dimensional dose distributions and depth–dose curves, owing to its very high spatial resolution and, for modern radiochromic films, near-water equivalence. Two main categories have been historically employed. Conventional radiographic films (silver–halide emulsions) were originally used for PDD determination by interleaving films between phantom layers or arranging them in stacks [5]. Although they enabled qualitative and semi-quantitative depth–dose measurements, their reliance on chemical development, pronounced energy dependence, and saturation at high doses have led to their progressive replacement by radiochromic films for quantitative work. Contemporary radiochromic films (e.g., Gafchromic EBT series) are self-developing: they darken in proportion to absorbed dose, have near-tissue-equivalent composition, and can be read out with standard flatbed scanners. For PDD measurements, radiochromic films may be placed at individual depths (one film per depth) or assembled into film stacks with intervening spacers to record an entire depth–dose curve in a single irradiation [39,70]. By analyzing the optical density of each layer, a high-resolution PDD can be reconstructed; using sub-millimeter spacing, detailed Bragg curves from single proton exposures can be obtained [37]. In both photon and ion beams, film-based techniques are widely used to benchmark Monte Carlo simulations and to characterize complex depth–dose distributions in situations where point detectors are impractical or excessively perturbative [71,72,73,74,75].

#### 3.2.1. Advantages

**Figure 5:**
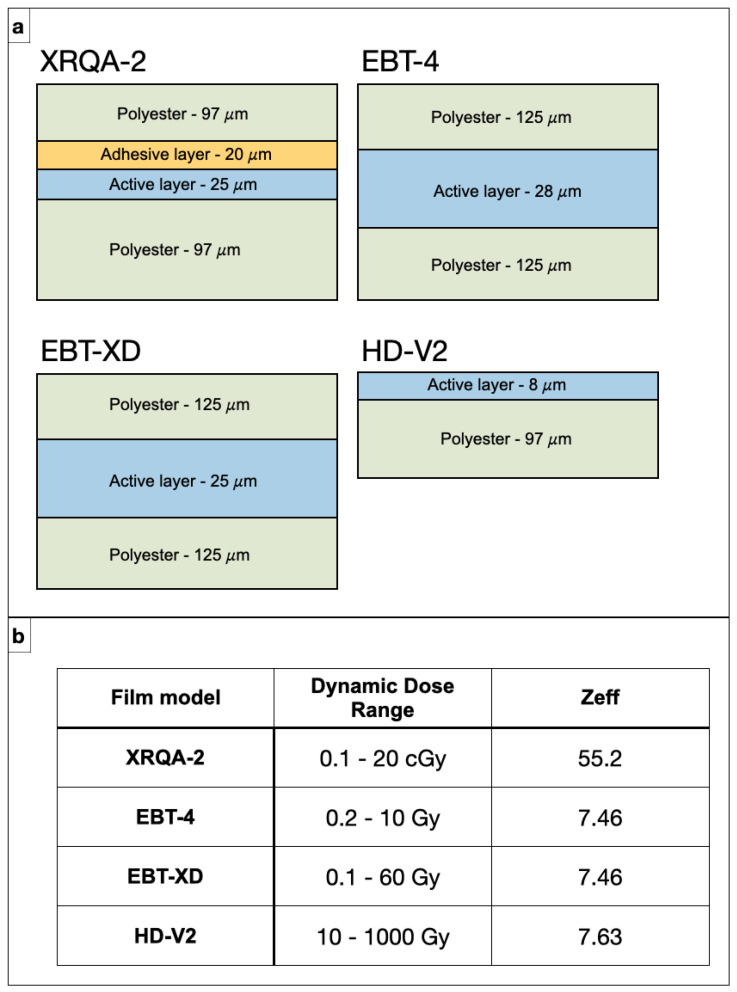
(**a**) Diagram of the Gafchromic film structure/dimensions: XRQA-2, EBT-4, EBT-XD and HD-V2 film model; (**b**) Available Gafchromic film models for radiation therapy and diagnostic radiology dose measurements with useful dose ranges and chemical compositions of sensitive layers.

The primary advantage of film dosimetry is its extremely high spatial resolution. The intrinsic resolution of the emulsion is on the micron scale, and typical scanner resolutions of 0.1–0.35 mm readily surpass what can be achieved with point detectors such as ionization chambers or diodes [37]. This enables a very detailed reconstruction of PDD shapes, including steep gradients, fine structure in Bragg peaks, and complex modulated fields. In the context of depth–dose measurements, radiochromic film stacks with sub-millimeter spacing can capture the entire PDD from a single irradiation, yielding finely sampled Bragg curves for proton and carbon-ion beams and making them particularly attractive for experimental studies and Monte Carlo validation [38,39,70]. Modern radiochromic films also offer near-tissue-equivalent composition, which reduces perturbations to the radiation field and simplifies the interpretation of measured optical-density profiles in terms of dose to water (see Figure 5) [5].

Their self-developing nature eliminates the need for chemical processing required by radiographic films, improving reproducibility and simplifying workflow. Because a large area can be exposed in a single shot, film is well-suited to mapping two-dimensional dose distributions simultaneously with depth–dose information, making it valuable for commissioning new beams, validating small-field PDDs, and checking complex treatment configurations or research setups where detailed spatial information is essential [5]. In such contexts, film measurements often serve as a high-resolution reference against which coarser but more routine detectors (e.g., ion chambers, arrays) are compared.

#### 3.2.2. Limitations

Films also present several important limitations that restrict their use in routine PDD acquisition. First, they are inherently passive detectors: there is no real-time readout, and radiochromic films require a post-irradiation stabilization period (typically of the order of ∼24 h) before scanning, during which the optical density continues to evolve [37]. Each piece of film is single-use, so repeated measurements over time incur material costs. Handling and readout are sensitive to environmental conditions (temperature, humidity, and ambient light) and to scanner-related issues such as non-uniform responses across the scanning bed and angular dependence, all of which must be carefully controlled to achieve accurate dose reconstruction [5,70]. In depth–dose measurements with film stacks, even small misalignments, variations in layer thickness, or air gaps between films and spacers can introduce systematic errors in the reconstructed PDD. Another major limitation is the energy- and LET-dependence of film response, particularly in proton and carbon-ion beams. In high-LET regions such as the Bragg peak and the nuclear-fragmentation tail, radiochromic films tend to under-respond relative to low-LET regions at the same physical dose, leading to an apparent distortion of the depth–dose curve unless dedicated LET-dependent correction strategies are applied [37,38,39]. While such corrections can be modeled or empirically derived, they add complexity and uncertainty, especially when films are used as quantitative reference standards. Because of these practical and physical limitations—passive nature, post-irradiation waiting time, single-use character, environmental and scanner sensitivities, and complex LET-dependent response—film dosimetry is less suited to day-to-day clinical PDD acquisition. Instead, it is most commonly employed for beam commissioning, research studies, and specific QA tasks where its unparalleled spatial resolution and ability to capture complex 2D and depth–dose distributions justify the additional effort and cost.

### 3.3. Solid-State Diode Detectors

Solid-state detectors based on semiconductor junctions have been employed for routine PDD measurements for decades, particularly in photon and electron beams. Conventional silicon diodes convert deposited energy into charge carriers within a small sensitive volume, producing a current proportional to the dose. Their compactness and electronic readout have made them standard tools in water-tank scanning systems, especially when high spatial resolution is required. Over time, other semiconductor materials such as synthetic single-crystal diamonds and wide-bandgap SiCs have been introduced, offering improved tissue equivalence, radiation hardness, and dose-rate performance for both conventional and advanced beams, including proton and carbon-ion therapy and UHDR conditions [29,30,76,77]. Additional semiconductor technologies (e.g., amorphous silicon, gallium nitride, silicon strip and pixel detectors) have been explored in more specialized roles such as flat-panel imaging or beam tracking, and may eventually contribute to high-accuracy PDD measurements where their practical constraints can be managed [2,78].

#### 3.3.1. Advantages

**Figure 6:**
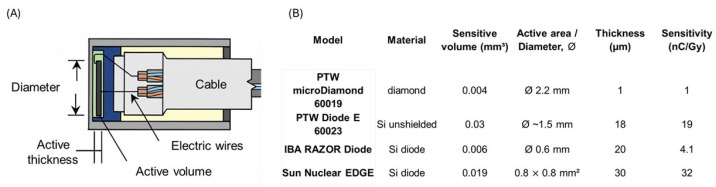
(**A**) Schematic cross-sectional view of a typical solid-state detector used for dosimetry. The detector consists of a small semiconductor sensitive volume embedded in a protective housing and connected to the readout electronics via electrical wires and a coaxial or triaxial cable. (**B**) Geometrical characteristics of commercially available solid-state detectors commonly used for clinical and research dosimetry, including sensitive volume, active area and thickness, and sensitivity [57,80,81].

Solid-state diode detectors provide high sensitivity with very small sensitive volumes (typically <1 mm^3^), which yields much finer spatial resolution than that achievable with standard ionization chambers [79] (see Figure 6).

This is particularly advantageous for scanning steep dose gradients, such as those found in the photon build-up region, in small-field beams, and around proton Bragg peaks. Because their signal is generated directly by charge collection in the semiconductor, diodes respond essentially instantaneously and offer real-time readout, making them well-suited to water-tank scanning, dynamic measurements, and fast QA procedures. In routine clinical practice, they are often cross-calibrated against a reference ion chamber at a given depth in water and then used to acquire PDD curves and profiles over the full depth range, leveraging their combination of sensitivity and spatial resolution. Beyond conventional silicon, other solid-state detectors extend these advantages. Synthetic single-crystal diamond detectors have become established tools in small-field and high-gradient dosimetry. Their near-tissue equivalence, extremely small sensitive volume, and high radiation tolerance make them suitable for photon, electron, and ion beams alike. Diamond detectors have demonstrated minimal dose-rate dependence and robust performance even at high instantaneous dose rates, including in FLASH-like irradiation regimes [82,83,84]. Wide-bandgap SiC has emerged as a particularly promising dosimetric material for proton and carbon beams and for UHDR conditions. SiC diodes exhibit low leakage current, excellent radiation hardness, fast charge collection, high linearity with dose, and near-dose-rate independence over a very broad range [27,29,31,44,76,85,86]. Experimental studies have shown that SiC diodes can reproduce proton PDDs with sub-millimeter accuracy in peak position when compared to reference plane-parallel ionization chambers, and maintain stable performance under large accumulated doses and in FLASH-like beams [27,30,77]. Other semiconductor technologies, including amorphous silicon, gallium nitride (GaN), and silicon strip/pixel detectors, offer high granularity and the possibility of integrating tracking or imaging capabilities with dosimetry [2]. In specific experimental or research contexts (e.g., flat-panel imagers, online beam tracking), these devices can provide detailed spatial information while still contributing to depth–dose characterization, underscoring the versatility of solid-state approaches in modern dosimetry.

#### 3.3.2. Limitations

The main limitations of conventional silicon diode detectors arise from energy dependence and radiation damage. In photon beams, diodes tend to over-respond to low-energy components, which can distort measurements in the build-up region and for large fields where scatter contributes a softer spectrum [2]. This can lead to an apparent enhancement of surface dose or an incorrect PDD shape if not properly corrected. Commercial designs with built-in filtration or compensation structures are used to flatten the energy response, but residual dependencies may remain and must be characterized during commissioning. Furthermore, silicon diodes are susceptible to radiation damage, particularly in mixed or high-neutron fields such as those encountered in proton and carbon-ion therapy; accumulated damage can alter their sensitivity and noise characteristics over time [48]. As a result, regular calibration checks and periodic replacement are necessary to ensure long-term stability. In proton beams, *p*-type silicon diodes used for depth–dose scanning exhibit LET-dependent response: they typically under-respond in the high-LET region of the Bragg peak, leading to an underestimation of the peak dose unless appropriate corrections are applied [2]. This LET dependence must be taken into account when using diodes as quantitative reference detectors, especially in proton and heavier-ion beams. While diamond and SiC detectors mitigate many of these issues through improved tissue equivalence, wide-bandgap properties, and enhanced radiation hardness, their deployment still requires careful calibration, and long-term clinical experience is comparatively limited. Other semiconductor systems, such as amorphous silicon, GaN, and silicon strip or pixel devices, face additional challenges related to radiation tolerance, stability, and practical implementation in routine clinical workflows. For instance, complex readout electronics, cooling or bias requirements, and the need for extensive quality-control procedures can limit their widespread adoption as primary PDD measurement tools. Consequently, although solid-state detectors—ranging from conventional silicon diodes to advanced diamond and SiC devices—offer clear advantages in sensitivity, spatial resolution, and, in some cases, dose-rate performance, they are generally used in conjunction with well-characterized ionization chambers. Cross-calibration against chamber-based reference dosimetry remains essential to manage energy and LET dependencies, radiation-damage effects, and other material-specific limitations.

### 3.4. Other Passive Dosimeters (TLDs, OSLDs)

Thermoluminescent dosimeters (TLDs) and optically stimulated luminescent dosimeters (OSLDs) are small passive sensors used to sample depth–dose distributions at discrete points. TLDs (e.g., LiF chips) and OSLDs (e.g., Al_2_O_3_:C) can be placed at various depths in a phantom, irradiated, and read out afterward, providing point-dose data. They have been used to verify PDDs where other detectors are impractical (e.g., in-vivo or irregular geometries). However, their limited spatial resolution, energy and dose-rate dependence, and labor-intensive handling make them better suited for point verification than complete PDD scanning [48].

## 4. Advanced and Emerging Technologies for PDD Measurement

### 4.1. Multi-Layer Ionization Chambers and Faraday Cup Arrays

**Figure 7:**
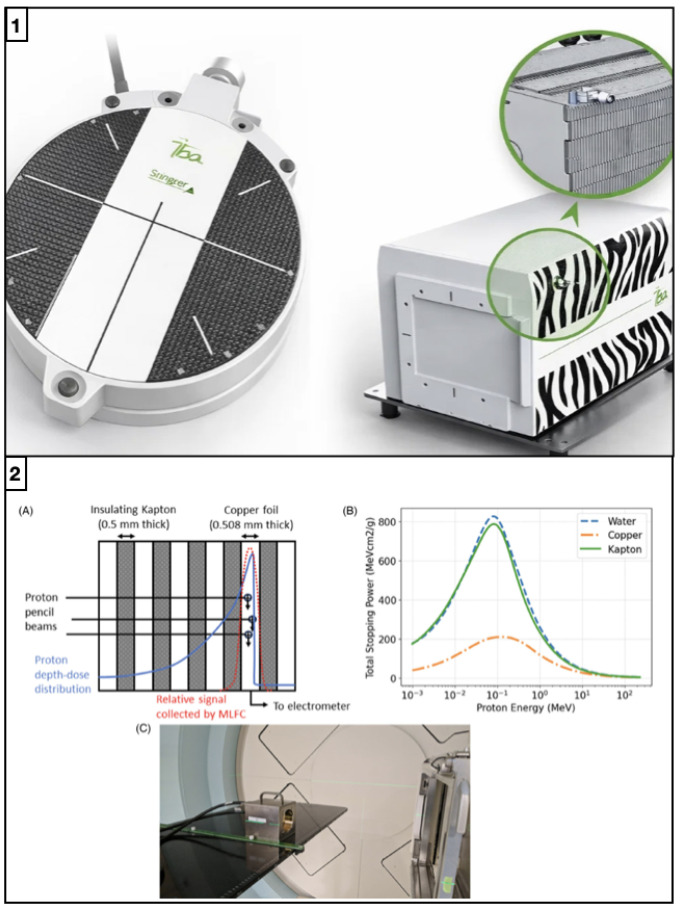
(**1**) Photo of the Stingray chamber (**left**), and photo of the Zebra detector (**right**) and sketch of the inner structure [88]. (**2**) (**A**) Schematic of MLFC and some of its copper and Kapton layers, showing how protons stopping at a copper foil are collected by the electrometer. The blue solid line depicts the proton Bragg peak, while the red dotted line depicts the relative signal collected by the layer. (**B**) Total stopping power of protons in water, copper, and Kapton obtained from PSTAR, NIST (National Institute of Standards and Technology). (**C**) Experimental setup of MLFC at NCCS. The Orfit indexing bar (Orfit Industries) ensures that the MLFC is parallel to the nozzle, which is parked at 90°. The MLFC is then connected to a 128-channel electrometer [89].

To accelerate depth–dose acquisition and reduce the reliance on time-consuming water-phantom scans, a class of multi-layer detectors that can measure an entire PDD curve with a single beam delivery has been developed. These systems consist of stacked active layers interleaved with absorber or water-equivalent materials, such that each layer samples the dose (or collected charge) at a different depth (see Figure 7). A single irradiation thus provides a discrete set of depth points spanning the full range of the beam. Among these devices, multi-layer ionization chambers (MLICs) have been widely investigated and deployed for proton and light-ion beam QA, while multi-layer Faraday cups (MLFCs) are primarily used in physics QA and research to derive beam energy spectra and range information [25,26,87].

#### 4.1.1. Advantages

MLICs consist of a stack of parallel-plate ionization chambers separated by water-equivalent material, with each chamber effectively sampling the dose at its corresponding depth in the phantom [90]. In a single irradiation, the signals from all layers provide a set of depth points that can be directly interpreted as a discretized PDD or Bragg curve. This configuration leads to a dramatic reduction in measurement time compared to conventional single-chamber water scans: instead of stepping a detector through many depths and repeating irradiations, the full depth–dose distribution is captured in one shot. This is particularly advantageous for proton and light-ion beam QA, where many Bragg peaks and modulation patterns must be verified. The typical layer spacing in MLICs is on the order of 1–2 mm, which is sufficient for most clinical range measurements and for verifying the overall shape and distal fall-off of therapeutic beams [88,91]. Because all layers are irradiated simultaneously, MLICs can also capture delivery fluctuations (e.g., from pencil-beam scanning or temporal instabilities) within a single acquisition, providing a snapshot of beam performance under realistic conditions. Their compact, self-contained design makes them convenient for routine QA procedures and for repeated checks of range and modulation without the logistical overhead of large water tanks [25,26]. MLFCs, in contrast, are designed primarily for high-precision range and energy measurements. They consist of alternating conductive and absorber layers, forming a stack that completely stops the incident charged-particle beam [89,92]. By measuring the charge collected in each conductive layer and relating it to the depth of energy deposition, an energy-loss profile and effective range can be reconstructed with very fine depth resolution. This makes MLFCs powerful tools for physics QA, accelerator tuning, and research applications where detailed information about the beam energy spectrum and stopping characteristics is needed.

#### 4.1.2. Limitations

Despite their efficiency, MLICs have intrinsic limitations related to spatial resolution and detector geometry. The discrete sampling imposed by the layer spacing results in a lower longitudinal resolution than can be obtained with finely stepped water-phantom scans. While this is generally adequate for clinical range verification, it can lead to partial smoothing of very sharp features, such as the most distal edge of a proton or ion Bragg peak. As a result, MLICs may slightly underestimate the steepness of the distal fall-off or fail to resolve subtle structures within complex modulation patterns, requiring complementary measurements or interpolation when very fine detail is needed [26]. In addition, the presence of multiple detector layers and absorber materials means that careful calibration is required to relate the measured signals to dose in water at the corresponding effective depths, and to account for any inter-layer variations in chamber response. MLFCs, although capable of extremely high depth resolution and detailed energy profiling, are inherently bulkier and more specialized than MLICs [89]. Because they are designed to stop the beam completely, they cannot be used in transmission under clinical treatment conditions and are therefore unsuitable for routine patient-specific QA. Their primary role remains in physics QA and research, such as beamline commissioning, energy-spectra characterization, and accelerator studies, rather than day-to-day clinical dosimetry. The requirement for sufficient shielding and space to accommodate the full stopping range of therapeutic ions further limits their integration into standard clinical workflows. Consequently, while both MLICs and MLFCs significantly speed up depth–dose and range measurements, their use is typically restricted to beam commissioning, periodic machine QA, and research applications, often in combination with more conventional dosimetric tools.

### 4.2. Scintillator-Based Detectors

Scintillation-based systems exploit materials that emit light in response to ionizing radiation and can provide real-time, high-resolution dosimetry. Depending on the geometry and readout scheme, they can sample dose at a single point, along a line, or over a 2D/3D volume. Liquid scintillator tanks, plastic scintillating probes and fibers, stacked scintillator detectors, 2D scintillating screens, and fiber-optic sensor arrays have all been proposed or implemented for depth–dose and PDD measurements in photon and particle beams [38,48,93,94,95]. These approaches are particularly attractive in situations requiring online range verification, high dose-rate capability (including FLASH), or minimally perturbing detectors that can be read out remotely via optical systems.

#### 4.2.1. Advantages

**Figure 8:**
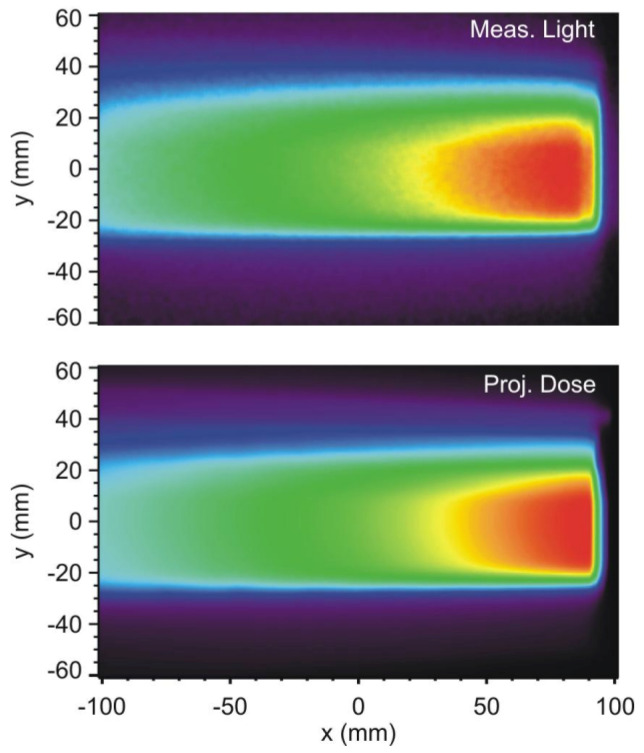
Comparison between the measured light and the predicted light from the projected dose. Both images are similar. However, the data within the measured light image (**top**) are noisier than within the forward-projected convoluted dose (**bottom**) [96].

Three-dimensional liquid scintillator detectors, such as those developed by Beddar’s groups, use large tanks of near-water-equivalent scintillating liquid imaged with cameras or photomultiplier arrays [93,96]. By recording the scintillation light distribution in real time, they can simultaneously capture proton range, lateral position, and intensity, enabling online monitoring of scanned or modulated beams (see Figure 8). Their composition is close to that of water, and with suitable reconstruction algorithms they can achieve sub-millimeter precision in proton range determination, making them powerful tools for research and advanced QA in particle therapy [93].

**Figure 9:**
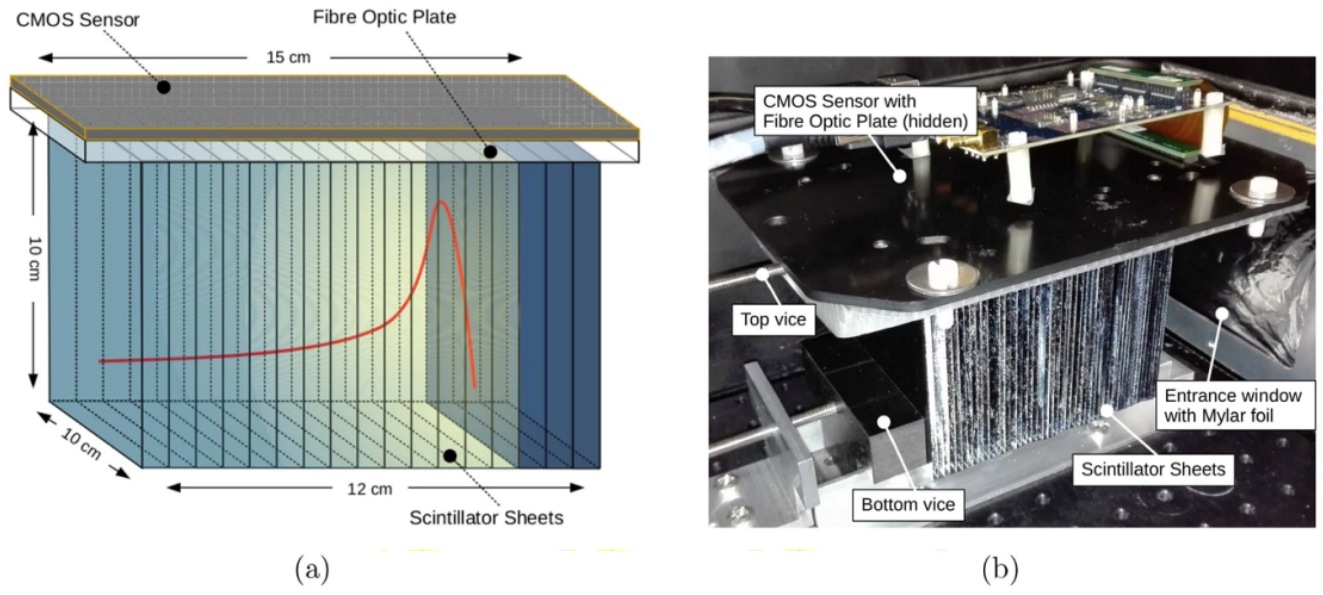
(**a**) Sketch and (**b**) photograph of the prototype scintillator-based range telescope with thin scintillator sheets and a CMOS sensor readout [97].

Plastic (solid) scintillators are widely used in dosimetry due to their near-water equivalence, fast temporal response, mechanical robustness, and ease of fabrication into small shapes [97,98]. Small scintillating probes or fiber tips can be mounted in scanning phantoms to provide point-by-point depth–dose measurements with very high spatial resolution, while stacked scintillator elements can sample multiple depths simultaneously (see Figure 9).

Plastic scintillators are largely dose-rate-independent and have been shown to perform well in high dose-rate beams, including FLASH-like conditions, where conventional ionization chambers may suffer from significant recombination losses [38,99]. Their prompt light output enables a true real-time readout with fast photodetectors, which is advantageous for dynamic delivery techniques. Scintillator stacks and 2D scintillating screens coupled to cameras or photomultipliers extend these benefits to multi-point and planar measurements. Stacked detector configurations can provide discrete depth sampling in a single irradiation, analogous to multi-layer ionization chambers but with potentially finer spatial resolution and optical readout. Recent work has demonstrated online Bragg-curve and energy reconstruction using scintillator stack detectors, showing that depth–dose and beam-energy characteristics can be inferred in real time from the light profiles in each scintillating layer [94]. Similarly, 2D scintillating screens can capture lateral dose profiles in a single acquisition and, by translating the screen along the beam axis (or by using stacked screens), can also provide depth-resolved information. Scintillating fiber arrays push this concept further: multiple fiber tips are positioned at predefined depths inside a phantom and their signals are routed to a multi-channel photodetector, so that a single irradiation yields dose readings at several depths—effectively producing a coarse PDD in one shot, as demonstrated by Jang et al. (2012) [95]. Although fiber-based approaches are still largely at the research stage for PDD-specific applications, they clearly illustrate the versatility of scintillator–fiber systems for future depth–dose measurements and online range verification.

#### 4.2.2. Limitations

Liquid scintillator detectors, despite their attractive real-time 3D imaging capabilities and water equivalence, pose several practical challenges. The scintillating solutions can be toxic or chemically hazardous, requiring careful handling, containment, and disposal procedures [93]. Their light yield is sensitive to quenching, temperature, and impurities, which necessitates careful control of environmental conditions and regular monitoring of optical properties. The optical readout itself is complex, typically involving cameras or photomultiplier arrays, precise geometrical calibration, and corrections for light attenuation, scattering, and background. The physical size and cost of full 3D liquid scintillator tanks further limit their use to specialized research or commissioning environments rather than routine clinical QA [93]. Plastic scintillators, while robust and dose-rate-independent, exhibit LET-dependent quenching in high-LET regions such as proton or ion Bragg peaks [38]. This leads to an under-response relative to the true absorbed dose in those regions, distorting the measured depth–dose curve if uncorrected. These factors can complicate long-term stability in clinical settings. Scintillator stacks and fiber arrays, although powerful for online Bragg-curve and profile measurements, also come with limitations. The depth resolution is constrained by the discrete thickness of the scintillating layers [94]. Optical cross-talk between layers, non-uniform light collection, and camera artifacts must be corrected to avoid systematic errors. Furthermore, the mechanical alignment with respect to the beam and the calibration of light-to-dose conversion can be non-trivial, especially in complex beam configurations. As a result, these systems are currently more common in experimental setups and advanced QA rather than as primary PDD measurement tools for routine clinical dosimetry [38,94].

### 4.3. Ionoacoustic Range Sensing

**Figure 10:**
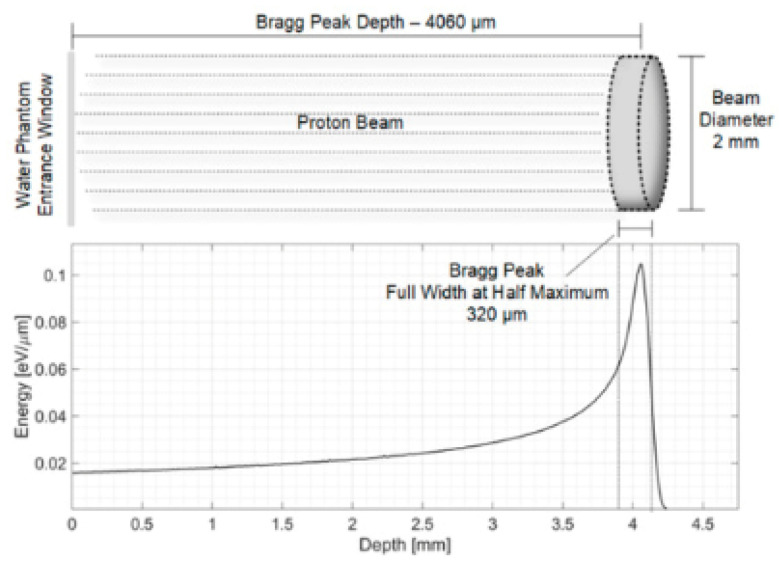
Experimental configuration adopted with an ionoacustic sensor to acquire 200 MeV proton Bragg peak [8].

Ionoacoustic detection exploits the acoustic waves generated by the rapid, localized energy deposition of charged particles at the Bragg peak. When a short, intense burst of energy is delivered to a small volume, a thermoelastic expansion occurs, launching pressure waves that can be detected by suitable acoustic sensors. By analyzing the time-of-flight and waveform of these signals, the position of the Bragg peak in a water-equivalent medium can be inferred with high precision. Experiments in water phantoms have demonstrated that ionoacoustic techniques can achieve sub-millimeter accuracy in determining proton range, using either piezoelectric transducers or optical hydrophones as receivers [100,101] (see Figure 10).

Recent work by Vallicelli et al. has further improved the practical performance of ionoacoustic systems by applying wavelet-based denoising algorithms to enhance the signal-to-noise ratio [8]. As a result, ionoacoustic methods have emerged as a promising candidate for real-time, non-invasive range monitoring in particle therapy.

#### 4.3.1. Advantages

The primary advantage of ionoacoustic range sensing lies in its direct sensitivity to the location of the Bragg peak via acoustic emission, rather than relying on secondary radiation or surrogate signals. Because the thermoelastic source is localized around the region of maximum energy deposition, the detected acoustic waveform encodes the position of the distal dose fall-off with high spatial resolution. Experimental studies in water phantoms have demonstrated sub-millimeter range precision using both piezoelectric and optical hydrophones, confirming the intrinsic potential of ionoacoustic methods for accurate range verification [8,101]. Another important strength is the possibility of real-time and non-invasive monitoring. Acoustic waves propagate at the speed of sound in tissue-equivalent media and can be detected outside the primary radiation field, allowing range information to be obtained during or immediately after beam delivery without disturbing the treatment. The use of optical or piezoelectric sensors placed at the phantom or patient boundary enables remote readout and avoids introducing additional high-Z materials into the beam path. Furthermore, advanced signal-processing techniques, such as the wavelet-based denoising proposed by Vallicelli et al., can significantly enhance the signal-to-noise ratio, making it easier to extract the Bragg peak position from weak and noisy measurements [8]. In principle, ionoacoustics can thus provide an in situ, online verification of range that complements or reduces the need for post-treatment imaging.

#### 4.3.2. Limitations

Despite its promise, ionoacoustic range sensing faces several challenges that currently limit its clinical deployment. The acoustic signals generated at therapeutic doses are intrinsically weak, especially when the energy deposition is distributed over extended times or larger volumes, so that the thermoelastic pressure rise is modest. As a consequence, high-sensitivity sensors and low-noise electronics are required, and the system remains vulnerable to ambient acoustic noise and mechanical vibrations, which can mask or distort the ionoacoustic waveform [101]. Complex clinical environments, with equipment motion and patient-related noise sources, exacerbate this problem and demand robust noise-reduction strategies. Although wavelet-based denoising and other advanced filtering methods have been shown to improve signal-to-noise [8], these algorithms add processing complexity and must be carefully validated to avoid bias in the reconstructed range. Acoustic coupling in realistic geometries presents another significant limitation. In simple water-phantom experiments, sensors can be optimally positioned and coupled to the medium; in patients, heterogeneous tissues, bone structures, and air cavities alter the propagation of acoustic waves and may introduce reflections, refractions, and attenuation that complicate the interpretation of the signals. Ensuring reliable coupling between the patient, coupling medium (e.g., gel), and transducers under clinical constraints (patient positioning, time limits, and sterility requirements) is non-trivial. Moreover, the need to maintain precise knowledge of the speed of sound along the propagation path introduces additional uncertainties. These technical and logistical hurdles must be overcome before ionoacoustic range sensing can progress from proof-of-concept demonstrations to widespread routine implementation in clinical particle therapy.

### 4.4. Prompt Gamma Techniques

**Figure 11:**
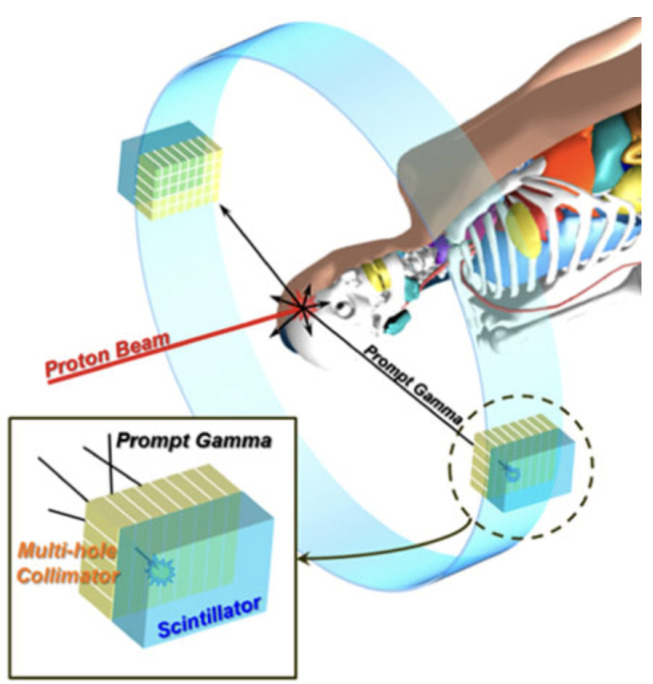
Schematic illustration of the working principle of the prompt gamma technique [102]. The incident particle beam is directed toward the patient/phantom, where nuclear interactions generate prompt gamma rays almost instantaneously along the beam path. The dashed circle highlights the region monitored for prompt gamma emission, while the black arrows indicate the detection geometry and signal collection path. In the inset, the different colors identify the main radiation components and materials involved: prompt gamma emission, the multi-slit collimator, and the scintillator detector.

Prompt gamma techniques infer proton or ion range by detecting nuclear prompt photons emitted almost instantaneously along the beam path during inelastic nuclear interactions. The resulting gamma rays are produced within nanoseconds of the primary ion traversal and escape the patient with minimal temporal delay, enabling range-sensitive information to be obtained during or immediately after irradiation (see Figure 11).

Various detector configurations have been developed to exploit this signal, including slit-camera systems and time-of-flight-based prompt gamma timing setups. Clinically tested slit-camera prompt gamma detectors, in particular, have demonstrated the feasibility of real-time range control in proton therapy, providing online verification of the beam’s distal fall-off during patient treatments [7].

#### 4.4.1. Advantages

The primary advantage of prompt gamma methods is their direct sensitivity to the longitudinal position of the beam along the irradiation path. Because prompt photons are emitted in close correlation with the depth-dependent nuclear interaction profile, the measured gamma emission pattern shifts when the proton or ion range changes, allowing detection of range deviations on a per-field or even per-spot basis [7]. This makes prompt gamma techniques particularly attractive for in-vivo range verification and adaptive radiotherapy, where the ability to detect millimeter-scale discrepancies between planned and delivered range can improve treatment safety and robustness. Slit-camera prompt gamma detectors have shown, in clinical trials, that it is possible to monitor the distal edge of the proton dose distribution in real time and to compare the measured prompt gamma profiles against pre-calculated references [7]. Deviations beyond a preset tolerance can be flagged during delivery, enabling rapid identification of anatomical changes, setup errors, or unexpected beam-path modifications. Since prompt gamma detection is non-invasive and does not require additional dose to the patient, it can be repeated at every fraction without altering the treatment plan. Furthermore, the use of collimated detection geometries and energy/time discrimination allows the suppression of a significant fraction of background radiation, making the extracted range signal sufficiently robust for clinical decision-making in selected scenarios [7]. Overall, prompt gamma systems offer a powerful, online, and beam-specific range monitoring capability that complements other, more indirect verification approaches.

#### 4.4.2. Limitations

Despite their demonstrated clinical feasibility, prompt gamma techniques face several limitations that currently restrict their widespread adoption. First, the instrumentation required is typically large, complex, and costly. Slit-camera systems, for example, rely on heavy collimators, high-efficiency scintillation or semiconductor detectors, and fast, high-throughput readout electronics to cope with the high instantaneous gamma fluxes encountered during proton or ion delivery [7]. These systems must be carefully integrated into the treatment room without interfering with patient positioning or gantry motion, which can be challenging in existing clinical infrastructures. Second, prompt gamma signals are inherently noisy and superimposed on substantial background from neutrons and scattered radiation, especially in high-energy or complex-treatment configurations. Extracting accurate range information requires sophisticated data-processing and statistical analysis, and the achievable spatial resolution and sensitivity depend strongly on the detector geometry, counting statistics, and treatment delivery mode [7]. Finally, current prompt gamma technologies are largely optimized for specific beam energies, gantry layouts, and clinical workflows, so transferring systems across centers or between treatment rooms can be non-trivial. Shielding requirements, calibration procedures, and quality assurance protocols add operational overhead, and the overall complexity can limit routine use outside specialized centers.

### 4.5. Gel Dosimetry

Gel dosimeters represent high-accuracy, largely water-equivalent systems that can, in principle, provide very detailed information on depth–dose distributions and absolute dose. Polymer and Fricke gels act as three-dimensional chemical dosimeters: the local radiation dose induces permanent chemical changes in the gel matrix, which can later be read out volumetrically using MRI or optical Computed Tomography (CT) to reconstruct the full 3D dose distribution, including PDDs [2].

#### 4.5.1. Advantages

Polymer and Fricke gel dosimeters provide a true three-dimensional recording of the delivered dose distribution [2,103]. After irradiation, the gel can be imaged by MRI or optical CT, yielding a volumetric map of dose with high spatial resolution throughout the entire irradiated volume. This makes it possible to extract PDDs along arbitrary lines of interest, as well as to analyze complex 3D dose patterns in modulated or non-coplanar beams, all from a single irradiation. Because the gel matrix can be formulated to be nearly water-equivalent, perturbations to the radiation field are minimal, and the resulting dose maps closely approximate dose to water. In the context of depth–dose measurements, gels are particularly attractive for commissioning novel treatment techniques or validating Monte Carlo simulations, where full 3D information and fine spatial detail are important [104].

#### 4.5.2. Limitations

Despite these strengths, gel dosimetry is limited by significant practical and technical challenges that hinder its use in routine PDD scanning. Polymer and Fricke gels are complex to manufacture and require stringent control of composition, oxygen content, and preparation conditions to ensure reproducible dose–response characteristics [2]. Their response is sensitive to environmental factors such as temperature and storage time, and may exhibit LET dependence, leading to under- or over-response in high-LET regions (e.g., near proton or ion Bragg peaks) unless carefully calibrated. The readout process itself—using MRI or optical CT—demands specialized, often expensive equipment, meticulous geometrical and dose calibration, and non-trivial image reconstruction and analysis. These requirements make gel dosimetry time-consuming and resource-intensive, restricting its use mainly to research, commissioning of new techniques, or quality audits rather than daily clinical PDD measurements.

## 5. Performance Comparison and Clinical Considerations

Given the wide range of detectors and techniques discussed in this review, a structured comparison is essential to clarify both their performance characteristics and the practical constraints associated with clinical deployment. The main evaluation criteria include spatial resolution, measurement accuracy, radiation hardness, dose-rate dependence, real-time capability, beam perturbation, and ease of use, as summarized in Table 1. In clinical practice, however, these categories are relevant only insofar as they support specific quantitative requirements, such as millimeter or sub-millimeter depth discrimination in steep gradients, stable response under repeated use, compatibility with traceable calibration workflows, and sufficiently fast acquisition for commissioning and routine quality assurance. For this reason, Table 1 provides a compact comparative overview, while Table 2 complements it by linking the main detector classes to the quantitative clinical targets they must satisfy and to the principal development paths that could strengthen their routine implementation. In clinical practice, detector selection is primarily driven by the measurement objective. For reference dosimetry and beam commissioning, water-phantom scanning with ionization chambers remains the preferred approach because it provides traceable absorbed-dose-to-water measurements and well-established long-term stability. However, the relatively large sensitive volume of ionization chambers can limit accuracy in regions characterized by steep dose gradients because of volume-averaging effects. When higher spatial resolution is required, such as for characterization of the build-up region, small-field dosimetry, or detailed analysis of the distal fall-off in particle beams, detectors with smaller sensitive volumes are generally preferred. In these situations, radiochromic films, diamond detectors, and small-volume semiconductor diodes can resolve fine spatial features with high precision, although their use typically requires careful calibration and corrections for energy or LET-dependent response. A critical aspect in the clinical adoption of emerging detector technologies concerns calibration and metrological traceability. While ionization chambers can be calibrated directly in terms of absorbed dose to water at primary or secondary standards laboratories according to international dosimetry protocols such as IAEA TRS-398 and AAPM TG-51, most alternative detector systems are typically calibrated indirectly. Semiconductor detectors (e.g., silicon, diamond, or silicon carbide diodes) are commonly cross-calibrated against a reference ionization chamber under well-defined beam conditions. Scintillator-based detectors require additional optical calibration procedures to account for light transport efficiency and possible quenching effects. For multi-layer ionization chambers and multi-layer Faraday cups, calibration is generally performed in terms of relative response or water-equivalent depth and verified against reference-depth–dose scans obtained with water-phantom measurements. These strategies provide practical traceability even when the detector itself cannot be directly calibrated in absorbed-dose-to-water units. For this reason, routine clinical workflows often rely on a hybrid measurement strategy that exploits the complementary strengths of different detector technologies. Ionization chambers are typically used for reference measurements and absolute dose normalization, multi-layer detectors or scintillator-based systems enable rapid acquisition of depth–dose or range-related information for periodic QA checks, and high-resolution methods such as film stacks are reserved for detailed investigations when fine spatial information is required. Rather than identifying a single optimal detector, the comparison highlights that each technology occupies a specific role within the broader clinical dosimetry workflow, where measurement accuracy, spatial resolution, robustness, and metrological traceability must be balanced according to the specific clinical task.

## 6. Current Challenges and Future Directions

Despite the significant progress in detector technology and measurement protocols, several fundamental challenges remain in achieving robust and clinically reliable PDD measurements across the full spectrum of modern radiotherapy modalities. These challenges arise not only from detector limitations, but also from the increasingly demanding delivery conditions encountered in contemporary clinical practice. First, the accuracy of depth–dose measurements is increasingly affected by steep dose gradients, small field sizes, and complex beam modulation. In such conditions, detector-related effects such as volume averaging, effective point-of-measurement uncertainties, and signal-chain stability can influence the determination of clinically relevant metrics, particularly in regions where range information is derived from the shape of the distal fall-off. Achieving high spatial resolution while maintaining metrological robustness therefore remains a central challenge for detector design. A second critical issue concerns detector response in mixed radiation fields. In proton and especially heavy-ion beams, the distal region of the PDD coincides with a rapid increase in LET and with the presence of secondary fragments. Many commonly used detectors exhibit some degree of LET dependence or quenching in these conditions, which may distort the reconstructed depth–dose distribution unless appropriate correction strategies or response models are applied. Developing detectors and analysis frameworks capable of maintaining a predictable response under varying radiation quality remains an active area of research. Another emerging challenge is the extension of dosimetric validation beyond homogeneous water phantoms. While water remains the reference medium for dosimetry, clinical treatments involve complex heterogeneous structures such as bone, air cavities, and implanted materials. Accurately verifying dose and range across such interfaces requires detectors capable of probing steep gradients with minimal perturbation, as well as modular phantom configurations that reproduce patient-relevant conditions. The increasing interest in ultra-high dose-per-pulse beams, including FLASH and other UHDR delivery schemes, further stresses the requirements placed on dosimetric systems. Under these conditions, detector linearity, dynamic range, and charge-collection stability become critical, and several conventional detectors may require significant correction factors or dedicated operating regimes. Ensuring traceable and reproducible measurements under these extreme irradiation conditions represents one of the major challenges for future dosimetry systems. Looking forward, the most impactful direction is not a single “universal” detector, but a convergence toward (i) improved standardization and inter-center comparability for modern detector classes and multi-layer systems, (ii) reduced dependence on ad hoc correction factors through intrinsically near-water-equivalent and low-perturbation designs, and (iii) response models that explicitly address LET/mixed-field effects. An ideal clinical PDD detector would therefore combine a small and well-defined effective measurement point, minimal beam perturbation, long-term stability and radiation hardness, real-time readout with wide dynamic range, and either a LET-insensitive response or a correction framework simple enough for routine clinical implementation. Among these characteristics, metrological traceability and long-term stability remain the most critical requirements for clinical use, as they ensure consistency with absorbed-dose-to-water standards used in reference dosimetry. When compared with currently available technologies, different detector classes approach this ideal from complementary perspectives. Ionization chambers remain the reference standard for traceable dose measurements, although their relatively large sensitive volume limits spatial resolution in steep dose gradients. Solid-state detectors could combine small sensitive volumes with excellent radiation hardness and therefore approach several characteristics of the ideal detector, particularly for high-resolution and high-dose-rate applications. Scintillator-based systems and scintillator stacks offer real-time readout and high spatial resolution, but their clinical use still requires careful calibration and correction for quenching and optical effects. These trade-offs explain why no single detector currently fulfills all requirements and why clinical PDD measurements typically rely on complementary detector technologies, each optimized for specific measurement tasks.

## Figures and Tables

**Table 1:** Comparative overview of detectors/techniques for depth–dose (PDD) and range-related measurements. The categories are intended as clinically oriented performance descriptors; representative quantitative targets relevant to routine implementation are summarized separately in Table 2.

Detector/Technique	Spatial Resolution	Measurement Accuracy	Radiation Hardness	Dose-Rate Dependence	Real Time	Beam Perturbation	Ease of Use
Water-tank ionization chamber (Farmer/pinpoint)	M (mm; volume averaging)	H (reference-grade)	H	H ^a^	Y (but scan-based)	M	M
Plane-parallel ionization chamber	M (∼1–2 mm)	H	H	H ^a^	Y	L–M	M
Radiochromic film (incl. stacks)	VH (≤0.3 mm readout; μm intrinsic)	H ^b^	N/A (single use)	L–M ^c^	N (offline)	L	L–M
Silicon diode (conventional)	H (sub-mm)	M–H ^b^	M	M–H ^a^	Y	L–M	H
Single-crystal diamond	H (sub-mm)	H	H	L	Y	L	M–H
SiC diode (wide bandgap)	H (sub-mm)	H ^b^	VH	L	Y	L	M
TLD/OSLD	L–M (discrete points)	M	M–H	M	N	L	L
MLIC (multi-layer ionization chamber)	M (layer spacing ∼1–2 mm)	H (range/PDD shape)	H	M–H ^a^	Y (one shot)	M	H
MLFC (multi-layer Faraday cup)	H (fine range/energy)	H	VH	L	Y	H (stops beam)	L
3D liquid scintillator tank	H (sub-mm range; volumetric)	M–H ^b^	M	L–M ^c^	Y	L	L
Plastic scintillator (point/fiber tip)	H (sub-mm)	M–H ^c^	H	L ^c^	Y	L	M–H
Scintillating screens/fiber arrays (multi-depth)	M–H (depth is discrete/ coarse)	M ^b^	H	L–M ^c^	Y	L–M	M
Ionoacoustic range sensing	H (sub-mm range)	M–H (range-focused)	H	M ^d^	Y ^d^	VL (non-invasive)	L–M
Prompt-gamma monitoring	M (range shift sensitivity)	M–H (range-focused)	H	N/A (not dose)	Y	VL (non-invasive)	L
Gel dosimetry (polymer/Fricke)	H (3D; voxel-limited)	H ^b^	N/A (chemical)	L–M ^c^	N (offline)	L	L

Scale:VH = very high, H = high, M = moderate, L = low, VL = very low; Y = yes, N = no. ^a^ Ionization-based detectors may require recombination/polarity corrections at very high dose-per-pulse or UHDR beams. ^b^ Accuracy depends strongly on calibration, setup, and correction factors (e.g., energy/LET effects; readout system). ^c^ Often weak intrinsic dose-rate dependence, but possible non-linearity/saturation and/or LET quenching depending on beam and method. ^d^ Real-time feasibility and dose-rate behavior depend on acoustic SNR and beam time structure. Representative quantitative clinical requirements include, depending on the task, millimeter or sub-millimeter depth discrimination, stable repeatability, traceable calibration, and acquisition times compatible with commissioning and routine QA. These aspects are summarized in Table 2.

**Table 2:** Main weaknesses of the principal detector classes, the corresponding quantitative clinical requirement, and the most relevant strengthening approach toward broader medical implementation.

Detector Class	Key Weakness	Clinical Quantitative Requirement	Strengthening Approach
Ionization chambers	Volume averaging; slow scans	Depth assignment in steep gradients at ∼1 mm level; stable traceable response for commissioning/reference dosimetry	Smaller sensitive volumes; faster scanning/positioning; improved UHDR recombination corrections
Radiochromic films	Offline workflow; LET/readout dependence	Sub-mm sampling for build-up/distal fall-off; reproducible readout for benchmarking and validation	Standardized scanning protocols; LET correction methods; improved registration in film stacks
Silicon diodes	Energy/LET dependence; radiation damage	Sub-mm real-time measurements with stable sensitivity under repeated use	Radiation-hard designs; validated correction factors; reference cross-calibration procedures
Diamond/SiC detectors	Limited standardization; limited routine clinical datasets	Sub-mm resolution, real-time response, long-term stability, robustness in high-dose-rate beams	Protocol-based calibration; multicenter validation; long-term clinical qualification, including FLASH-like conditions
MLIC/MLFC	Discrete depth sampling	Reliable range/PDD reconstruction with ∼1–2 mm or finer effective sampling for routine QA	Reduced layer spacing; improved interpolation/deconvolution; systematic comparison with water-tank reference scans
Scintillator-based systems	Optical calibration complexity; quenching/non-linearity	Fast high-resolution acquisition with quantitatively stable optical response	Robust optical calibration pipelines; quenching corrections; compact clinically deployable readout systems
Prompt-gamma/ionoacoustic methods	Indirect range information; not direct absorbed-dose measurement	Online range verification with mm or sub-mm sensitivity and sufficient signal-to-noise ratio	Improved detection efficiency/SNR; integration with conventional dosimetry workflows
Gel/passive point dosimeters	Cumbersome preparation/readout; pointwise or offline workflow	3D verification or local validation with reproducible spatial registration	Simplified preparation/readout; use in end-to-end validation rather than routine PDD acquisition

## Data Availability

No new data were created or analyzed in this study.
